# Metabolic syndrome in haemodialysis patients: prevalence, determinants and association to cardiovascular outcomes

**DOI:** 10.1186/s12882-020-02004-3

**Published:** 2020-08-13

**Authors:** Arnaud Delautre, François Chantrel, Yves Dimitrov, Alexandre Klein, Olivier Imhoff, Clotilde Muller, Nicole Schauder, Thierry Hannedouche, Thierry Krummel

**Affiliations:** 1 Service de Néphrologie et Dialyse, Hôpitaux Universitaires de Strasbourg, Strasbourg, France; 2 Faculté de Médecine, Université de Strasbourg, Strasbourg, France; 3 Service de Nephrologie, Centre Hospitalier Emile Muller de Mulhouse, Mulhouse, France; 4 Association pour l’Utilisation du Rein Artificiel en Alsace (AURAL), Strasbourg, France; 5 Service de Nephrologie, Centre Hospitalier Général de Haguenau, Haguenau, France; 6 Service de Nephrologie, Centre Hospitalier Général de Colmar, Colmar, France; 7 Service de Nephrologie, Clinique Sainte-Anne, Strasbourg, France; 8 Observatoire Régional de la Santé d’Alsace (ORSAL), Strasbourg, France

**Keywords:** Metabolic syndrome, Haemodialysis, Waist circumference, Major cardiovascular events, Epidemiology

## Abstract

**Background:**

In the general population, metabolic syndrome (MetS) is predictive of major adverse cardiovascular events (MACE). Waist circumference (WC), a component of the MetS criteria, is linked to visceral obesity, which in turn is associated with MACE. However, in haemodialysis (HD) patients, the association between MetS, WC and MACE is unclear.

**Methods:**

In a cross-sectional study of 1000 HD patients, we evaluated the prevalence and characterised the clinical predictors of MetS. The relationship between MetS and its components, alone or in combination, and MACE (coronary diseases, peripheral arteriopathy, stroke or cardiac failure), was studied using r**eceiver operating characteristics (**ROC) curves and logistic regression.

**Results:**

A total of 753 patients were included between October 2011 and April 2013. The prevalence of MetS was 68.5%. Waist circumference (> 88 cm in women, 102 cm in men) was the best predictor of MetS (sensitivity 80.2; specificity 82.3; AUC 0.80; *p* <  0.05). In multivariate analysis, MetS was associated with MACE (OR: 1.85; 95CI 1.24–2.75; *p* <  0.01), but not WC alone. There was a stronger association between the combination of abdominal obesity, hypertriglyceridaemia and low high-density lipoprotein cholesterol with MACE after exclusion of impaired fasting glucose and hypertension.

**Conclusions:**

MetS is frequent and significantly associated with MACE in our haemodialysis cohort and probably in other European dialysis populations as well. In HD patients, a new simplified definition could be proposed in keeping with the concept of the “hypertriglyceridaemic waist”.

## Background

Obesity is a significant risk factor for diabetes, high blood pressure and coronary heart disease in the general population and contributes to overall mortality worldwide. In dialysed patients, the relationship between body mass index (BMI) and mortality is more complex, with a U or L relationship [1, 2]. The maximum risk of death is strongly associated with a low BMI while overweight and obesity are associated with either a modest increase in mortality rates or no increase at all [1–3]. These paradoxical results, coined “reverse epidemiology”, have also been observed in other diseased populations including heart failure or cancer, particularly in observational short-term follow-up studies [4].

BMI is a global parameter that does not dissociate abdominal adiposity (visceral white fat) which has deleterious metabolic effects, from muscle mass and brown fat which are protective factors [5]. Indeed, in the general population, several studies, notably the INTERHEART study, found a closer relationship between waist circumference (WC), which refers to abdominal obesity, and cardiovascular mortality, comparatively to BMI. After adjustment for diabetes and high blood pressure, BMI no longer had a predictive value on cardiovascular mortality [6]. Similarly, in the NHANES III study, WC was an independent predictor of cardiovascular mortality [7]. In another study, it was also the single parameter explaining the relationship between obesity and cardiovascular morbidity [8].

In the general population, studies have found that the presence of the metabolic syndrome (MetS), defined by the combination of abdominal fatness, lipid abnormalities, hyperglycaemia and hypertension, was a better indicator of cardiovascular death than obesity alone [9]. Occasionally, MetS has been considered as a more robust cardiovascular risk factor than diabetes alone [10, 11]. MetS and diabetes were firmly associated, both in the general population [13] and dialysis patients. However, in diabetic patients, the metabolic syndrome is further associated with an increased cardiovascular risk [12]. The risk of post-transplant diabetes was also found to be predicted by pre-transplantation MetS [14].

In the haemodialysis population, there are only few data on the relationship between MetS and cardiovascular morbidity and mortality. Most previous studies have focused on the prevalence of MetS. Still, these were small cohorts [15–26], originating from countries around the Mediterranean Sea or in Asia with a low baseline prevalence of MetS.

Besides, most studies in dialysis patients have been aimed at comparing the definition of metabolic syndrome according to various criteria (NCEP ATP III 2001, IDF 2005 and HMetS criteria 2009). In the present study, we used the latest 2009 classification (HMetS 2009), which is deemed as the most predictive of mortality in haemodialysis patients [15].

The primary objective of this study was to assess the prevalence of MetS in a large regional population of chronic haemodialysis patients, and to identify the predictors of MetS or its components, including waist circumference, either alone, or in combination.

The secondary objective was to establish the relationship between MetS and its components, alone or in association, with cardiovascular history and comorbidities and to compare the strength of this association with that of BMI and diabetes.

## Methods

This observational cross-sectional study was conducted using data from the REIN registry (Nephrology Information and Epidemiological Network) nested in the Alsace region (Northeastern France) and was approved by the Comité Consultatif sur le Traitement de l’Information en matière de Recherche dans le domaine de la Santé (CCTIRS) and the Commission Nationale de l’Informatique et des Libertés (CNIL).

The following demographic data and clinical characteristics were collected in all patients treated by chronic haemodialysis in Alsace (*n* = 1000) between October 2011 and April 2013:

1. Age, gender
2. Weight, height, waist circumference (WC) and body mass index (BMI). WC was measured in each centre by the same operator according to the World Health Organisation (WHO) method [27]. The reference value in the analysis represents the average of 3 consecutive measurements.
3. Fasting serum HDL-cholesterol (HDL), LDL-cholesterol (LDL), total cholesterol and triglycerides (TG)
4. History of high blood pressure or current antihypertensive treatment
5. Fasting plasma glucose, HbA1c
6. Diagnosis or history of diabetes
7. Smoking, current or past
8. Serum albumin
9. History of cardiovascular disease
  Stroke
  Congestive heart failure (CHF)
  Coronary heart disease (CHD):
    Myocardial infarction
    Presence of coronary stenosis
    Coronary revascularization with angioplasty or coronary artery bypass surgery
  Congestive heart failure (CHF).
  Peripheral arterial disease (PAD) (ordered stages 3 and 4)
10. Ongoing statin treatment
11. Dialysis vintage

All participating patients gave their informed consent to participate in the study. Data were extracted from computerised medical records Medware (Sined, Italy), shared by all dialysis centres of the region.

Metabolic syndrome (MetS) was defined according to the 2009 criteria (HMetS: « Harmonizing the Metabolic Syndrome ») [28]. The HMetS criteria suggest using either the 2005 or 2001 thresholds as a choice. We chose the highest, namely those of NCEP ATP III, recognised as adapted for European patients:

1. Waist circumference (MsWC):
  > 102 cm in men
  > 88 cm in women
2. HDL-cholesterol (MsHDL)
  < 0.50 g/L (< 1.29 mmol/L) in women
  < 0.40 g/L (< 1.03 mmol/L) in men [29]
3. Triglycerides (MsTG) ≥ 1.5 g/L (≥ 1.7 mmol/L)
4. Fasting blood glucose (MsGlc) ≥ 1.00 g/L (≥ 5.6 mmol/L)
5. High blood pressure (MsHBP): Blood pressure values were not directly available and tend to exhibit cyclic variations in HD patients. Therefore, the “hypertension” criterion was defined by the declaration of a history of hypertension in the patient’s record, or an ongoing antihypertensive treatment.

The presence of 3 criteria or more among the five was used to define the metabolic syndrome according to HMetS 2009 criteria [28].

Major adverse cardiovascular events (MACE) were defined according to the REIN registry criteria [30], by the presence of at least one of the following items in the patient’s history:

- CHD (defined by a history of bypass surgery or angioplasty, or coronary artery disease documented by a stress test).

- Peripheral arterial disease (PAD) stage 3 or 4 (PAD 3–4), defined as stage 3: rest pain; stage 4: trophic disorders or amputation.

- Stroke with permanent sequelae (Stroke). Transient ischaemic attacks (TIAs), which have more uncertain diagnoses, were not included.

- Congestive heart failure (CHF).

### Statistical analyses

Results are expressed as means ± standard deviation for quantitative variables and compared with Student’s t test when following a normal distribution, and by the Mann-Whitney test for non-normal distributions. The normality of the distribution was tested graphically together with the Shapiro-Wilk test.

Qualitative variables are described as the frequency and percentage of each of the modalities and were compared using the Chi-square test.

The relationship between BMI and waist circumference was studied using the Bravais-Pearson correlation coefficient (normal distribution of the data).

Since diabetes may affect the presence of MetS and MACE, the analyses were stratified in both diabetic and non-diabetic patients.

The association between components of the metabolic syndrome and the full metabolic syndrome was studied using ROC curves and calculating sensitivities, specificities, positive and negative predictive values.

The association of waist circumference with the metabolic syndrome was also tested by logistic regression with adjustment for other parameters potentially associated with MetS including diabetes, haemoglobin A1c, BMI, smoking, age, gender, statin treatment and serum albumin.

Relationships between metabolic syndrome and history of cardiovascular events and cardiovascular comorbidities were studied in univariate analyses and then multivariate analysis. For the multivariate analysis, a logistic regression model was adjusted for diabetes, LDL-cholesterol, smoking status, age, sex, BMI, statin use and serum albumin. The procedure was performed using a step-by-step backward selection (backward elimination). Parameters included in the multivariate analysis were those with a *P* <  0.20 in the univariate analysis. Only serum albumin was “forced” into the multivariate analysis despite a *P* > 0.20 given its acceptance as a significant prognostic factor in the dialysis population. Univariate and multivariate analyses were also repeated for studying the relationship between risk factors and coronary heart disease, PAD stage 3–4, stroke and CHF individually.

The relationships between MACE and the various associations of the MetS criterion, but always including MsWC, were studied using the same univariate and multivariate models as described above.

The nonparametric Wilcoxon test was used to study the number of cardiovascular events between groups (abnormal distribution of the data).

All statistical analyses were performed with the STATA v14 software (Statacorp LLC, Texas, USA).

## Results

The results were analysed for 753 patients (75.3% of the initial cohort) for whom all data were available.

**Fig. 1:**
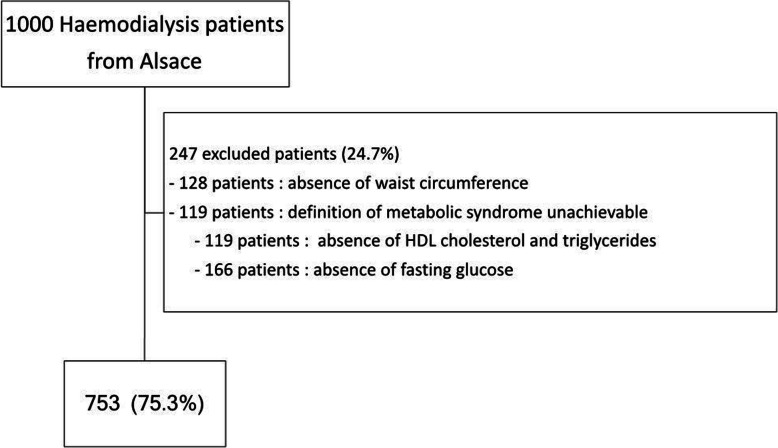
Study flowchart

The missing data mostly consisted of WC measurements, not available in 128 patients (refusal of patients to participate or impossibility of standing upright for the measure), absence of fasting HDL and TG in 119 patients and absence of fasting blood glucose in 166 patients (Fig. 1).

For the 753 studied patients, the main demographic data are summarised in Table 1 and the presumed nephropathies leading to end-stage kidney disease in Table 2. There were 436 men (58%) and 317 women (42%). Mean patient age was 65.5 ± 0.5 years (65 ± 0.7 years for men, 66.5 ± 0.7 years for women) while body mass index was 27.2 ± 0.2 kg/m^2^ for the whole population and 26.7 ± 0.3 kg/m^2^ and 27.9 ± 0.4 kg/m^2^for men and women, respectively. Low BMIs (< 20 kg/m2) were observed in 8.5% of the cohort. Prevalence of overweight (BMI 25–29.99) and obesity (BMI ≥ 30) was 32.1 and 28.3%, respectively.

1. Prevalence and determinants of the metabolic syndrome

**Table 1:** Main clinical features and distribution of the components of the metabolic syndrome in the 753 haemodialysis patients with or without metabolic syndrome

		MetS+	MetS-	*p*
Prevalence. n (%)		516 (68.5)	237 (31.5)	
Gender, n (%)				
men		282 (54.7)	154 (65.0)	**< 0.01**
women		234 (45.3)	83 (35)	
Age, years		66.6 ± 0.5	63.3 ± 1.1	**< 0.01**
Body height, cm		165.2 ± 0.4	165.7 ± 0.6	0.50
Weight, kg		79.2 ± 0.8	64.4 ± 0.9	**< 0.01**
Dialysis vintage, years		5.0 ± 0.2	6.4 ± 0.5	**< 0.01**
Serum albumin, g/L		37.7 ± 0.2	37.9 ± 0.3	0.60
BMI, kg/m2		29.0 ± 0.3	23.4 ± 0.3	**< 0.01**
BMI > 30, n (%)		195 (38)	12 (8)	**< 0.01**
BMI 25–30, n (%)		184 (36)	50 (21)	**< 0.01**
BMI < 20, n (%)		13 (3)	51 (22)	**< 0.01**
Diabetes, n (%)		299 (58)	44 (19)	**< 0.01**
HbA1c, %		6.7 ± 0.2	5.8 ± 0.1	**0.01**
Statin use, n (%)		329 (64)	121 (51)	**< 0.01**
Smoking, n (%)		228 (54)	103 (53)	0.80
WC, cm		107.4 ± 0.6	90.2 ± 0.8	**< 0.01**
Fasting glucose, g/L		1.3 ± 0.03	1.0 ± 0.03	**< 0.01**
HDL, mmol/L		1.0 ± 0.02	1.4 ± 0.03	**< 0.01**
with statin, mmol/L		1.0 ± 0.02	1.4 ± 0.03	**< 0.01**
without statin, mmol/L		1.1 ± 0.02	1.4 ± 0.03	**< 0.01**
TG, mmol/L		2.4 ± 0.06	1.3 ± 0.03	**< 0.01**
with statin, mmol/L		2.5 ± 0.06	1.3 ± 0.03	**< 0.01**
without statin, mmol/L		2.1 ± 0.06	1.3 ± 0.03	**< 0.01**
LDL, mmol/L		3.1 ± 0.1	3.5 ± 0.2	**0.03**
with statin, mmol/L		2.9 ± 0.1	2.9 ± 0.1	**0.63**
without statin, mmol/L		3.5 ± 0.1	4.1 ± 0.2	**0.06**
MsWC, N (%)		414 (80)	42 (18)	**< 0.01**
men		197 (70)	16 (10)	**< 0.01**
women		217 (93)	26 (31)	**< 0.01**
MsHBP, n (%)		483 (74)	174 (26)	**< 0.01**
MsHDL, n (%)		333 (67)	24 (10)	**< 0.01**
men		187 (66)	13 (8)	**< 0.01**
women		146 (62)	11 (13)	**< 0.01**
MsTG, n (%)		330 (69)	36 (16)	**< 0.01**
MsGlc, n (%)		423 (85)	93 (43)	**< 0.01**

*MetS+* Patients with metabolic syndrome; *MetS-* Patients without metabolic syndrome; *WC* Waist circumference; *TG* Triglycerides; *MsWC* Waist circumference > 102 cm in men or > 88 cm in women; *MsHBP* High blood pressure or antihypertensive drugs; *MsHDL* HDL cholesterol < 0.50 g/L in women or <  0.40 g/L in men; *MsTG* TG ≥1.5 g/L; *MsGlc* Fasting glucose ≥1.00 g/L.

**Table 2:** Distribution of presumed causes of kidney diseases in the 753 haemodialysis patients with or without metabolic syndrome

	MetS+	MetS-
Diabetes, n(%)	205 (40)	25 (10)
Glomerulonephritis, n(%)	69 (13)	63 (27)
Hypertension, n(%)	44 (9)	22 (9)
Unknown, n(%)	48 (9)	25 (10)
Polycystic, n(%)	33 (6)	18 (8)
Pyelonephritis, n(%)	16 (3)	14 (6)
Vascular, n(%)	7 (1)	0
Autre, n(%)	94 (18)	70 (30)
Total	516 (100)	237 (100)

*MetS* Metabolic syndrome

Of the 753 patients, 68.5% were diagnosed with MetS according to the HMetS definition, with a prevalence of 64.7% in men and 73.8% in women (*p* <  0.01).

A total of 456 patients (60.6%) had MsWC. The mean WC was 102.0 ± 0.6 cm. The MsWC criterion was present in 48.8% of men and 76.7% of women, with an MsWC of 103.1 ± 0.7 cm and 100.5 ± 0.9 cm, respectively.

In obese or overweight patients, 96.7 and 74.4% had MsWC, respectively. The relationship between BMI and WC was highly significant (*r* = 0.84 *p* <  0.001), and equally observed in both genders.

Among the 753 patients, 343 (45.6%) were diabetic, of whom 28.3% were obese and 19.4% overweight. In people with diabetes, WC was 109 ± 0.8 cm and the MsWC criterion was found in 77% of these patients (*n* = 264). The difference in WC between diabetic and non-diabetic patients (96 ± 0.7 cm) was highly significant (*p* <  0.001).

In the whole cohort, the prevalence of the other MetS criteria, namely MsHDL, MsTG, MsGlc and MsHBP was 50.3, 51.5, 72 and 87.3%, respectively.

The association between the MetS components and MetS is shown in Table 3. There were 414 patients (55%) with both MsWC and MetS while 80% of patients with MetS had MsWC (sensitivity: 80%), including 197 (70%) and 217 (93%) in men and women, respectively. In patients without MetS, 195 did not have MsWC (specificity: 82%). The prevalence of MetS in patients with MsWC was 90.8%.

**Table 3:** Association between MetS components and the presence or absence of MetS

		MsWC	MsHBP	MsHDL	MsTG	MsGlc
Total population	Sensitivity	80.23	93.6	69.81	68.89	84.6
	Specificity	82.28	26.8	89.66	84.48	57.14
	PPV	90.79	73.52	93.28	90.16	81.98
	NPV	65.66	65.62	59.09	56.81	61.69
Men	Sensitivity	69.86	93.97	71.37	70.61	84.56
	Specificity	89.61	23.28	91.33	84.00	54.68
	PPV	92.49	69.19	93.50	88.52	78.50
	NPV	61.88	67.92	64.62	62.07	64.41
Women	Sensitivity	92.74	93.16	67.91	66.82	84.65
	Specificity	68.67	32.53	86.59	85.37	61.54
	PPV	89.30	79.56	92.99	92.36	86.55
	NPV	77.03	62.79	50.71	49.30	57.83

*MetS* Metabolic syndrome. *PPV* Positive predictive value. *NPV* Negative predictive value. *MsWC* Waist circumference > 102 cm in men or > 88 cm in women. *MsHBP* High blood pressure or antihypertensive drugs. *MsHDL* HDL cholesterol < 0.50 g/L in women or <  0.40 g/L in men. *MsTG* TG ≥1.5 g/L. *MsGlc* Fasting glucose ≥1.00 g/L.

**Fig. 2:**
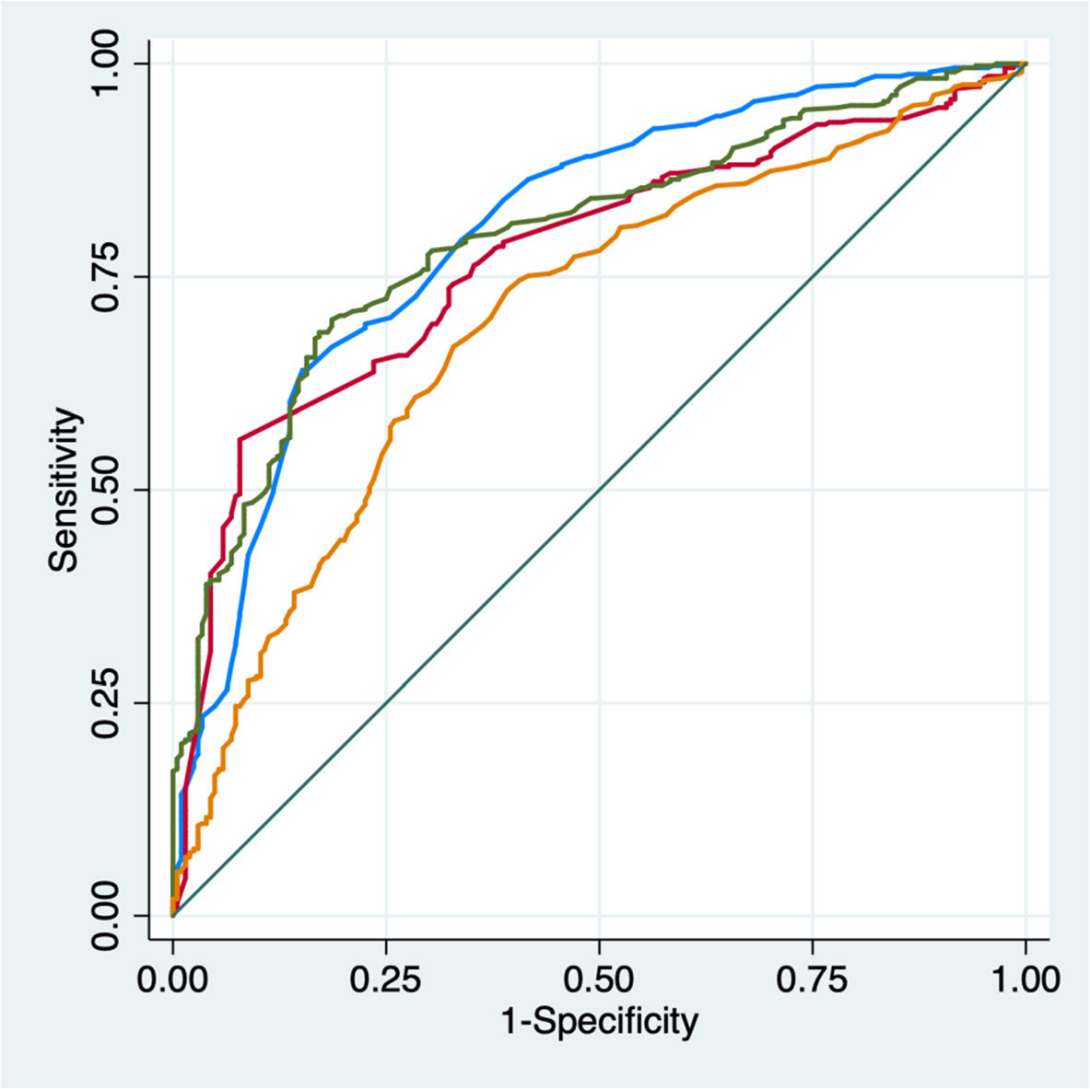
ROC curves of the association between isolated MetS parameters and MetS. Legend: **—** Waist circumference, AUROC = 0.80. **—** HDL cholesterol, AUROC = 0.77. **—** TG, AUROC = 0.797. **—** Fasting glucose, AUROC = 0.70. AUROC: Area Under the ROC

ROC curves were plotted to study the relationship between the various individual parameters and MetS, from which areas under the curve (AUC) were calculated (Fig. 2). The following findings, in descending order, were observed:

### For both genders

WC (0.80), TG (0.797), HDL (0.77), Glc (0.70). With WC = TG (*p* = 0.5), WC & TG > HDL (*p* <  0.05), HDL > Glc (*p* <  0.05).

### In men

HDL (0.83), WC (0.8012), TG (0.8011), Glc (0.68). With HDL > WC (*p* <  0.05), WC = TG (*p* = 0.5), WC & TG > Glc (*p* <  0.05).

### In women

WC (0.84), TG (0.79), HDL (0.76), Glc (0.74). With WC = TG (*p* = 0.5), WC & TG > HDL (*p* <  0.05), HDL > Glc (*p* <  0.05).

MetS was very highly prevalent in obese diabetics (97%) and was found in “only” 48% of non-diabetic and non-obese patients.

The association of waist circumference with metabolic syndrome was tested with adjustment for diabetes, HbA1c, body mass index, tobacco use, age, sex, statin treatment and serum albumin. WC remained significantly associated with MetS after adjustment (OR 1.10. *p* <  0.001; 95CI 1.07–1.13). Diabetes and male gender were also significantly associated with MetS with an OR of 3.51 (2.0–6.23) and 0.38 (0.20–0.72), respectively (*p* <  0.05 for both).

BMI was conversely not significantly associated with MetS (OR 0.96; 95CI 0.87–1.07; *p* = 0.52) (Table s1).

2) Metabolic syndrome and cardiovascular complications

In the 753 patients, the distribution of MACE was: 244 patients (32.4%) with CHD, 68 patients (9%) with stage 3 or 4 PAD, 114 patients (15.1%) with stroke, and 140 patients (18.6%) with congestive heart failure. In the whole cohort, 379 (50.3%) had at least one cardiovascular complication, and 225 (29.9%), 123 (16.3%), 29 (3.8%), and 2 (0.27%) had one, two, three or four CV complications, respectively.

**Fig. 3:**
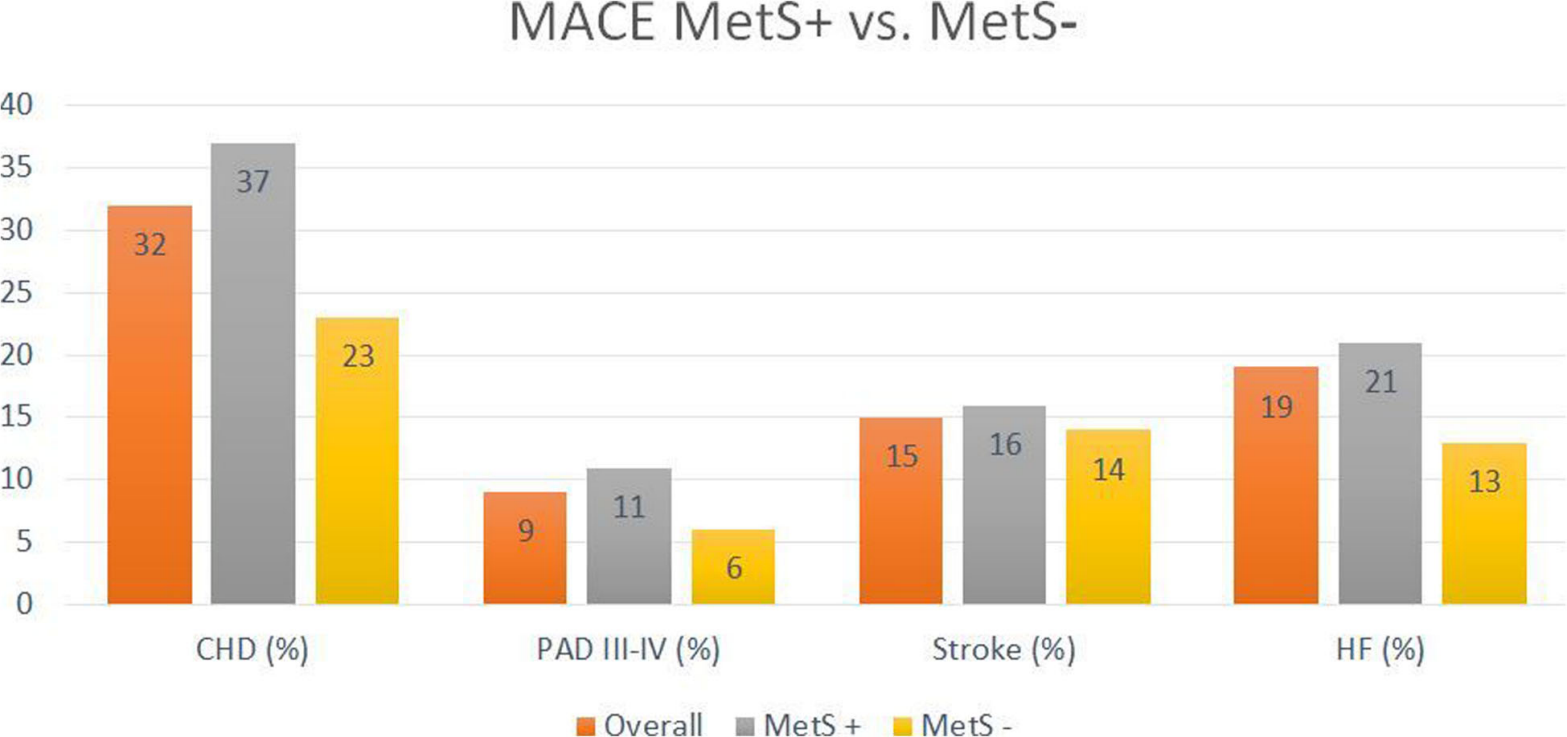
Distribution of MACE subtypes according to the presence or absence of metabolic syndrome. MetS: Metabolic syndrome. MACE: major adverse cardiovascular events. CHD: coronary heart disease. PAD III-IV: peripheral arterial disease, stage III or IV according to the classification of Leriche and Fontaine. HF: Heart failure. Significant difference (*P* < 0.05) between MetS+ and MetS- for CHD, PAD III-IV and HF; not significant for Stroke

Among the 516 patients with MetS, 56% exhibited at least one MACE compared to 38% for the group of patients without MetS. The individual subtypes of MACE based on the presence or absence of MetS are listed in Table s2 and Fig. 3.

The global MACE criterion was associated with MetS in univariate (OR 2.13; 95CI 1.56–2.92; *p* <  0.01) as well as multivariate analysis (OR 1.85; 95CI 1.24–2.75; *p* <  0.01) after adjustment for diabetes, LDL-cholesterol, smoking, age, sex, BMI, statin treatment and serum albumin (Table 4).

**Table 4:** Multivariate logistic regression of risk factors of overall major cardiovascular events

	OR	*p*	95% CI
**MetS**	1.85	**< 0.01**	1.24–2.75
LDL (g/L)	1.07	0.14	0.98–1.17
**Diabetes**	1.58	**0.02**	1.08–2.32
**Smoking**	1.78	**< 0.01**	1.24–2.55
**Age (year)**	1.03	**< 0.01**	1.01–1.04
BMI (kg/m2)	0.98	0.22	0.94–1.01
**Statin use**	2.71	**< 0.01**	1.87–3.93
Serum albumin (g/L)	0.99	0.75	0.96–1.03
Male	1.07	0.76	0.71–1.60

*MetS* Metabolic syndrome; *LDL* LDL cholesterol; *BMI* Body mass index. *OR* Odds ratio

In univariate analysis, MetS was associated with coronary artery disease, congestive heart failure and PAD 3–4 with a OR of 2.0 (1.4–2.8; *p* <  0.001), 1.9 (1.2–2.9; *p* = 0.005) and 1.9 (1.0–3.5; *p* = 0.04), respectively. In multivariate analysis, MetS was significantly associated with coronary artery disease (OR 1.92; 95CI 1.22–3.01; *p* <  0.01) and heart failure (OR 2.35; 95CI 1.40–3.99; *p* <  0.01), but not with PAD 3–4 or stroke (Table 5).

**Table 5:** Multivariate logistic regression of risk factors according to specific major cardiovascular event

	CHD			PAD III-IV			STROKE			CHF		
	OR	*p*	95% CI	OR	*p*	95% CI	OR	*p*	95% CI	OR	*p*	95% CI
MetS	1.92	**< 0.01**	1.22–3.01	0.79	0.58	0.33–1.81	0.90	0.71	0.51–1.60	2.35	**< 0.01**	1.40–3.99
LDL (g/L)	1.09	0.07	0.99–1.20	0.82	0.06	0.66–1.01	0.95	0.43	0.84–1.08	1.09	0.10	0.98–1.21
Diabetes	1.76	**< 0.01**	1.18–2.63	5.90	**< 0.01**	2.78–12.5	1.89	**0.01**	1.14–3.14	1.32	0.26	0.81–2.15
Smoking	1.55	**0.04**	1.01–2.37	1.84	0.11	0.87–3.90	1.76	**0.03**	1.07–2.90	1.38	0.20	0.84–2.28
Age (year)	1.03	**< 0.01**	1.02–1.05	1.02	0.17	0.99–1.05	1.03	**0.01**	1.01–1.05	1.02	0.09	1.00–1.03
BMI (Kg/m2)	1.00	0.83	0.96–1.04	0.97	0.39	0.92–1.04	0.95	**0.04**	0.91–0.998	0.97	0.54	0.95–1.03
Statin use	2.94	**0.02**	1.93–4.47	2.53	**0.02**	1.18–5.44	2.59	**< 0.01**	1.47–4.55	1.22	0.41	0.76–1.97
Serum albumin (g/L)	1.00	0.82	0.97–1.04	1.01	0.63	0.96–1.08	1.02	0.43	0.97–1.07	0.96	**0.01**	0.92–0.99
Male	1.72	**0.02**	1.10–2.67	0.64	0.24	0.30–1.36	0.84	0.54	0.47–1.48	0.69	0.14	0.42–1.14

*MetS* Metabolic syndrome; *LDL*: LDL cholesterol; *BMI* Body mass index; *CHD* Coronary heart disease. *PAD 3–4* Stage III or IV peripheral arterial disease; *CHF* Congestive heart failure

An association between MetS and cumulative MACE number (*p* <  0.001) was found with a mean of 0.84 ± 0.04 MACE in patients with MetS compared to 0.55 ± 0.5 in the group of patients without MetS.

#### Components of the metabolic syndrome and MACE

The association between the individual components of the metabolic syndrome and cardiovascular complications was also analysed.

There was a significant association between MsWC and the number of cardiovascular comorbidities with a mean of 0.80 ± 0.04 MACE in the group with MsWC versus 0.67 ± 0.05 in the group without MsWC (*P* = 0.049). However, MsWC was only associated with coronary artery disease (OR 1.6; 95CI 1.2–2.2; *p* <  0.05) and PAD 3–4 (OR 1.8; 95CI 1.0–3.1; *p* <  0.05) in both univariate and multivariate analysis (Table s3). In multivariate analysis, diabetes was significantly associated with cardiovascular events including coronary artery disease, PAD 3–4 and stroke (OR 1.76 [95CI 1.18–2.63], *p* <  0.001; 5.90 [95CI 2.78–12.5], *p* <  0.001; and 1.89 [95CI 1.14–3.14], *p* <  0.001), but no significant association with heart failure.

The other components of the MetS taken individually were not associated with MACE in multivariate analysis, except for MsHDL (OR 1.56; 95CI 1.06–2.30; *p* <  0.02).

However, in multivariate analysis, there was a significant relationship between MACE and a combination of MsWC + MsTG (OR 1.75; 95CI 1.09–2.81; *p* = 0.02) and a combination of MsWC + MsTG + MsHDL (OR 2.11; 95CI 1.26–3.54; *p* <  0.01). In contrast, the combinations of 4 or 5 MetS criteria were not associated with MACE in multivariate analysis. These data are summarised in Table 6 and Figure s1.

**Table 6:** Cumulative effects of metabolic syndrome components in predicting MACE in univariate and multivariate analysis. Multivariate analysis adjusted for diabetes, LDL-cholesterol, smoking, age, sex, BMI, statin use and serum albumin

		Univariate analysis			Multivariate analysis		
	N/Nt(%)	OR	*p*	95% CI	OR	*p*	95% CI
**1 criterion**							
MsWC	456/753 (60.6)	1.32	0.063	0.99–1.77	1.31	0.3	0.79–2.18
MsTG	366/711 (51.5)	1.46	**0.01**	1.09–1.96	1.17	0.46	0.78–1.75
MsHDL	357/709 (50.4)	1.77	**< 0.01**	1.32–2.39	1.56	**0.02**	1.06–2.30
MsHBP	657/753 (87.3)	1.82	**< 0.01**	1.17–2.82	1.76	0.049	1.0–3.10
MsGlc	516/717 (72.0)	2.67	**< 0.01**	1.90–3.75	1.25	0.42	0.73–2.16
**2 criteria**							
MsWC + MsTG	254/720 (35.3)	1.73	**< 0.01**	1.27–2.36	1.75	**0.02**	1.09–2.81
MsWC + MsHDL	247/714 (34.6)	1.71	**< 0.01**	1.25–2.33	1.53	0.079	0.95–2.46
MsWC + MsHBP	413/753 (54.85)	1.38	**0.03**	1.04–1.85	1.48	0.093	0.94–2.32
MsWC + MsGlc	361/741 (48.7)	1.83	**< 0.01**	1.37–2.45	1.4	0.2	0.84–2.34
**3 criteria**							
MsWC + MsTG + MsHDL	169/716 (23.6)	1.93	**< 0.01**	1.36–2.75	2.11	**< 0.01**	1.26–3.54
MsWC + MsHDL + MsHBP	226/714 (31.7)	1.78	**< 0.01**	1.29–2.45	1.53	0.079	0.95–2.46
MsWC + MsHDL + MsGlc	200/706 (28.3)	2.05	**< 0.01**	1.47–2.86	1.39	0.21	0.83–2.33
MsWC + MsTG + MsHBP	227/720 (31.5)	1.67	**< 0.01**	1.22–2.30	1.39	0.15	0.89–2.16
MsWC + MsTG + MsGlc	211/712 (29.6)	1.88	**< 0.01**	1.35–2.60	1.28	0.34	0.77–2.12
MsWC + MsGlc + MsHBP	327/743 (44.0)	1.89	**< 0.01**	1.41–2.53	1.49	0.11	0.92–2.42
**4 criteria**							
MsWC + MsGlc + MsHBP + MsHDL	185/708 (26.1)	2.16	**< 0.01**	1.53–3.05	1.59	0.085	0.94–2.69
MsWC + MsGlc + MsHBP +MsTG	189/714 (26.5)	1.85	**< 0.01**	1.32–2.59	1.2	0.5	0.72–2.00
MsWC + MsGlc + MsHDL +MsTG	145/712 (20.4)	1.96	**< 0.01**	1.35–2.85	1.49	0.15	0.87–2.55
MsWC + MsTG + MsHBP + MsHDL	152/716 (21.2)	2,00	**< 0.01**	1.39–2.90	1.62	0.15	0.83–3.16
**5 criteria**							
MsWC + MsGlc + MsHDL+ MsHBP +MsTG	132/714 (18.5)	2.1	**< 0.01**	1.42–3.11	1.58	0.11	0.9–2.8

*MsWC* Waist circumference > 102 cm in men or > 88 cm in women. *MsHBP* High blood pressure or antihypertensive drugs. *MsHDL* HDL cholesterol < 0.50 g/L in women or < 0.40 g/L in men. *MsTG* TG ≥ 1.5 g/L. *MsGlc* Fasting glucose ≥1.00 g/L. *N/NT* Number of patient with the criteria (MsWC, MSTG …) / number of patients with available data. *OR* Odds ratio

Low serum albumin was associated with heart failure (OR 0.96; 95CI 0.92–0.99; *p* = 0.01). Only MetS and low serum albumin were associated with heart failure in multivariate analysis.

In diabetic patients, MetS was not associated with MACE in multivariate analysis (OR 2.28; 95CI 0.99–5.24; *p* <  0.05) while in non-diabetic patients, MetS was associated with MACE in multivariate analysis (OR 1.88; 95CI 1.16–3.04; *p* = 0.01).

## Discussion

In the present study, the prevalence of the metabolic syndrome and its components, measured individually or in combination, was analysed in a comprehensive cohort of haemodialysed patients in Alsace. The analysis also assessed whether the metabolic syndrome or various combinations of its components were associated with cardiovascular history and comorbidities, as compared with BMI and diabetes.

Our multicentre study in 753 patients comprised a representative proportion of the regional haemodialysis population (75%). Of note, half of the non-included patients were excluded because WC measurements were deemed unfeasible due to the inability to stand up. To our knowledge, the present patient population represents the largest studied cohort of patients treated on maintenance haemodialysis and dealing with MetS.

Irrespective of the criteria used, a high prevalence of MetS was observed, present in more than half of the patients. Abdominal obesity (increased waist circumference) was also prevalent, particularly in women. These data reflect the exceedingly high prevalence of diabetes and obesity in the general Alsatian population, which also explains diabetic nephropathy as the leading cause of end-stage renal failure in this region [30].

As expected, the relationships between obesity, diabetes, metabolic syndrome and high WC were strong. The metabolic syndrome is a complex entity involving several criteria for its definition, which has evolved over time. The present study used the criteria from the latest 2009 classification (HMetS 2009), which appear better suited to haemodialysis patients [15].

In our population, however, not all criteria had the same positive predictive value (PPV), as high WC, hypo-HDLaemia and hypertriglyceridaemia were identified as the most specific. Unsurprisingly, an increased waist circumference, a marker of abdominal adiposity, was the single most potent factor associated with the metabolic syndrome in our cohort. In contrast, the “high blood pressure” criteria was less specific, likely because hypertension could result from a number of other causes in this dialysis population, including arterial stiffness and volume overload.

In this cross-sectional study, more than 50% of the patients exhibited at least one cardiovascular comorbidity, confirming the high burden of cardiovascular comorbidities in this population. However, in the present haemodialysis cohort, metabolic syndrome was strongly and independently associated with major cardiovascular events (coronary artery disease, peripheral arteriopathy, stroke or congestive heart failure), with this association persisting after adjustment for other cardiovascular risk factors, including diabetes and BMI. This association was even more robust with certain specific complications, namely coronary heart disease and congestive heart failure.

The strength of this association may, however, have been underestimated due to the cross-sectional nature of the analysis. Indeed, patients with MetS, who faced a higher risk, could have died prematurely after starting haemodialysis. Also, most of the patients in whom measurement of WC was impossible were more likely to be arteriopathic amputees or post-stroke hemiplegics.

As expected, diabetes was strongly associated with MACE in our cohort. In the general population, the relationship between diabetes and metabolic syndrome is strong [31]. In our cohort, however, the harmful effect of the metabolic syndrome persisted after adjustment for diabetes, demonstrating an independent prognostic impact. An analysis excluding diabetics further confirmed a significant association between the metabolic syndrome and MACE, confirming previous reports in the general population [32]. Nevertheless, in the subgroup analysis studying diabetic patients, there was no significant association between metabolic syndrome and MACE. A study with more power would probably prove a statistical link between MS and MACE in diabetic patients. In contrast, neither WC alone nor BMI were associated with cardiovascular history in multivariate analysis, thereby corroborating the data observed for BMI in the general population.

More surprisingly, statin treatment was associated with a 2.7-fold higher risk of cardiovascular complications. In our study, 60% of the patients were on statin treatment, including a majority of patients with MetS. Unlike LDL-cholesterol, both HDL-cholesterol and triglycerides were ostensibly relatively uninfluenced by the statin treatment. Although randomised trials have failed to find any apparent benefits of statins in dialysis patients, a direct deleterious effect of statin is seemingly unlikely, this paradox likely reflecting an indication bias, since statins are prescribed preferentially in individuals at high risk or with a history of cardiovascular complications.

When analysing the cardiovascular events individually, the association with metabolic syndrome was significant for coronary heart disease and congestive heart failure but not for peripheral arteriopathy or stroke.

There are sparse data in the literature on the metabolic syndrome in dialysis patients, with data applicable to European populations being particularly scarce. Moreover, studies have yielded conflicting results. A prospective Tunisian study in 200 haemodialysis patients found an association between the metabolic syndrome and cardiovascular events [18]. Another Iranian retrospective study conducted in 300 haemodialysis patients showed an increased risk of coronary heart disease associated with the metabolic syndrome [19]. In contrast, the prospective study of Tsangalis et al. in 102 patients in Greece did not find an association between metabolic syndrome and MACE [20]. These authors suggested a survival bias in explaining these results since patients with metabolic syndrome could have been the first to die before inclusion in the study. In a prospective Chinese study, 157 patients exhibited no association between metabolic syndrome, CVD, and mortality [21]. In these two last studies, MetS was associated with a better nutritional status, a possible confounding factor. Of note, we did not find any difference in serum albumin levels in patients with or without the metabolic syndrome.

### Components and simplified MetS

The current definition of the metabolic syndrome is somewhat complex as it involves parameters requiring a strictly fasting measurement (blood glucose, HDL-cholesterol, triglycerides) or potentially associated with high variability (blood pressure). This may hinder the evaluation of MetS and raises the question of whether a simple measurement of waist circumference could provide similar prognostic information.

In the present study, we found no significant association between high waist circumference (MsWC) and history of MACE, after adjustment for diabetes and BMI. These results are inconsistent with those of a prospective Italian study of the CREDIT registry (Calabria Registry of Dialysis and Transplantation) [16], which analysed the effect of waist circumference on global and cardiovascular mortality in 537 haemodialysis patients. In this south-Italian cohort, the prevalence of obesity and MsWC was much lower (12 and 39% respectively) comparatively to the present cohort. While BMI was inversely correlated with mortality, the increase in WC was directly associated with an increased risk of overall and cardiovascular death. Moreover, after adjusting for other risk factors, a 10 cm higher WC was found to increase overall mortality and cardiovascular mortality by 26 and 38%, respectively. Among all the other risk factors (cardiovascular history, age, duration of dialysis, haemoglobin, CRP), only the presence of diabetes increased the risk of overall (OR 2.73) and cardiovascular (OR 2.88) mortality. Unfortunately, this Italian study did not investigate the prognostic value of the full-blown metabolic syndrome on cardiovascular mortality.

In Japan, an association between directly CT-measured abdominal adiposity and cardiovascular disease, including stroke and coronary artery disease was observed in a small cohort of haemodialysis patients [17–26].

These findings, however, have not been consistent. In Taiwan, another prospective study in 91 patients found more cardiovascular events associated with the metabolic syndrome and increased WC [28], but no association with mortality [22]. These authors also found that abdominal obesity was correlated with PAD, independently of other factors related to MetS and inflammation [23].

BMI is a global parameter that does not dissociate abdominal adiposity (visceral white fat), which has harmful metabolic effects, from muscle mass and brown fat which, conversely, are protective factors [5, 33]. However, WC does not differentiate visceral adiposity which is more strongly associated with the risk of diabetes [34, 35], dyslipidaemia [36], hypertension [37] and CV complications [5, 38], from subcutaneous adiposity, which is potentially more metabolically benign. Visceral obesity can be better quantified using CT scans of L4-L5 [5, 24, 25, 39], although this technique is poorly suitable for epidemiological studies. Some authors have proposed the concept of “hypertriglyceridaemic waist”, associating a high waist circumference and an increase in serum triglycerides. This syndrome has even been found more strongly correlated with visceral obesity [5, 35] and cardiovascular diseases [40].

In the dialysis population, hypertension is prominent, although its association with cardiovascular morbidity and mortality remains controversial [41]. Since hypertension results from multiple mechanisms in haemodialysis patients [42], its integration into the definition of the metabolic syndrome is hence debatable.

Furthermore, our findings show that the combinations of MsWC + MsTG or MsWC + MsTG + MsHDL were those most significantly associated with cardiovascular complications. Indeed, the addition of a 4th or 5th parameter (either blood glucose or high blood pressure) did not yield any additional prognostic information. Our results thus support the use of the “hypertriglyceridaemic waist” as a marker of cardiovascular risk in haemodialysis patients.

Finally, only MetS and low serum albumin were associated with heart failure in multivariate analysis. In haemodialysis patients, low serum albumin is frequent and strongly associated with both cardiac and overall mortality [43–45]. Nevertheless, the significance of low serum albumin is multiple, including malnutrition but also chronic inflammation (IL6-dependent liver synthesis), as well as haemodilution related to volume overload.

The strengths of the study include the relatively large homogeneous cohort, representative of a contemporary European population. We also used a harmonised coding system nested in the regional REIN registry. Finally, the WC measurement was standardised across all participating centres.

Some limitations of the study should bear in mind. Although the cohort was representative, nearly 25% of the patients were excluded from the analysis, some of whom could have provided valuable information. Collection of MACE was performed retrospectively at the time of initiation of maintenance dialysis and inclusion in the REIN registry, and yearly thereafter. On the other hand, chart history during pre-dialysis follow-up was available in a vast majority of patients. Finally, this study was cross-sectional with the inherent limitations due to surviving bias, and statistical associations cannot be ascertained as being causative.

## Conclusions

In this cross-sectional study, the metabolic syndrome was common in our regional haemodialysis cohort and was strongly correlated with diabetes and obesity.

A robust relationship was found between the metabolic syndrome and cardiovascular history, a link which was not observed with BMI or waist circumference alone.

The “simplified metabolic syndrome”, combining high WC + hyperTG + hypoHDL, was more effective in predicting CV history, a finding consistent with the concept of “hypertriglyceridaemic waist” and may suggest a redefinition of the syndrome, specific to haemodialysed populations.

A large prospective study in haemodialysis patients aimed at confirming the associations between metabolic syndrome and cardiovascular events and mortality, is currently underway.

## Supplementary information

**Additional file 1 Table s1.** Multivariate logistic regression of predictive risk factors of metabolic syndrome.

**Additional file 2 Table s2.** Distribution of MACE according to the presence or absence of metabolic syndrome (MetS+ vs. MetS-).

**Additional file 3 Table s3.** Association between individual MACE subtypes and MsWC in univariate analysis.

**Additional file 4 Figure s1.** Forest plots of MetS, MetS without BPH, and cumulative effect of components of the metabolic syndrome in predicting MACE in multivariate analysis.

## Data Availability

This study was conducted using data from the REIN registry (Nephrology Information and Epidemiological Network) in the Alsace region (Northeastern France).

## Abbreviations

BMI: Body mass index
CHD: Coronary heart disease
CHF: Congestive heart failure
HD: Haemodialysis
HF: Heart failure
HMetS: Harmonizing the metabolic syndrome
LDL: LDL-cholesterol
MetS: Metabolic syndrome
MetS-: Patients without metabolic syndrome
MsGlc: Fasting glucose ≥1.00 g/L
MsHBP: High blood pressure or antihypertensive drugs
MsHDL: HDL cholesterol < 0.50 g/L in women or < 0.40 g/L in men
MsTG: TG ≥1.5 g/L
MsWC: Waist circumference > 102 cm in men or > 88 cm in women
MACE: Major adverse cardiovascular events
NPV: Negative predictive value
OR: Odds ratio
PAD: Peripheral arterial disease
PAD 3–4: Stage III or IV peripheral arterial disease
PPV: Positive predictive value
REIN registry: Nephrology information and epidemiological network registry
TIAs: Transient ischaemic attacks
TG: Triglycerides
WC: Waist circumference
WHO: World health organisation
