# Supplementary material for: Metabolic syndrome in haemodialysis patients: prevalence, determinants and association to cardiovascular outcomes
Source: BMC Nephrol. 2020 Aug 13;21:343. doi: 10.1186/s12882-020-02004-3 (PMC7427285; doi:10.1186/s12882-020-02004-3)
Supplement: Supplementary file 1 — Additional file 1 Table s1. Multivariate logistic regression of predictive risk factors of metabolic syndrome. [file 12882_2020_2004_MOESM1_ESM.docx]

**Table s1 : Multivariate logistic regression of predictive risk factors of metabolic syndrome.**

|  | OR | p | 95% CI |
| --- | --- | --- | --- |
| Age | 0.99 | 0.46 | 0.97-1.01 |
| Serum albumin (g/L) | 1.01 | 0.72 | 0.95-1.07 |
| BMI (kg/m2) | 0.96 | 0.52 | 0.87-1.07 |
| **Diabetes N(%)** | **3.51** | **<0.001** | **2.0-6.23** |
| HbA1c (%) | 1.16 | 0.34 | 0.86-1.57 |
| Statin N(%) | 0.92 | 0.79 | 0.50-1.69 |
| Smoking N (%) | 0.82 | 0.54 | 0.43-1.55 |
| **WC (cm) N = 753** | **1.10** | **<0.001** | **1.07-1.13** |
| **Male** | **0.38** | **0.003** | **0.20-0.72** |

WC: waist circumference. OR: Odds ratio.
