# Supplementary material for: Metabolic syndrome in haemodialysis patients: prevalence, determinants and association to cardiovascular outcomes
Source: BMC Nephrol. 2020 Aug 13;21:343. doi: 10.1186/s12882-020-02004-3 (PMC7427285; doi:10.1186/s12882-020-02004-3)
Supplement: Supplementary file 2 — Additional file 2 Table s2. Distribution of MACE according to the presence or absence of metabolic syndrome (MetS+ vs. MetS-). [file 12882_2020_2004_MOESM2_ESM.docx]

**Table s2: Distribution of MACE according to the presence or absence of metabolic syndrome (MetS+ vs. MetS-)**

|  | MetS + | MetS - | p |
| --- | --- | --- | --- |
| MACE n (%) | 290 (56) | 89 (38) | **< 0.01** |
| Mean number of MACE | 0.8 | 0.6 | **< 0.01** |
| CHD n (%) | 190 (37) | 54 (23) | **< 0.01** |
| PAD 3-4 n (%) | 54 (11) | 14 (6) | **0.04** |
| Stroke n (%) | 81 (16) | 33 (14) | 0.53 |
| HF n (%) | 110 (21) | 30 (13) | **0.01** |

MetS: metabolic syndrome; MACE: major adverse cardiovascular events; CHD: coronary heart disease; PAD 3-4: peripheral arterial disease, stage 3 or 4 according to the classification of Leriche and Fontaine; HF: heart failure.
