# Supplementary material for: Metabolic syndrome in haemodialysis patients: prevalence, determinants and association to cardiovascular outcomes
Source: BMC Nephrol. 2020 Aug 13;21:343. doi: 10.1186/s12882-020-02004-3 (PMC7427285; doi:10.1186/s12882-020-02004-3)
Supplement: Supplementary file 3 — Additional file 3 Table s3. Association between individual MACE subtypes and MsWC in univariate analysis. [file 12882_2020_2004_MOESM3_ESM.docx]

**Table s3: Association between individual MACE subtypes and MsWC in univariate analysis**

|  | MsWC | | |
| --- | --- | --- | --- |
|  | HR | P | 95% CI |
| CHD | 1.61 | **< 0.01** | 1.17-2.21 |
| PAD 3-4 | 1.79 | **0.04** | 1.03-3.08 |
| Stroke | 0.74 | 0.14 | 0.50-1.11 |
| HF | 1.12 | 0.42 | 0.80-1.71 |

MsWC: waist circumference > 102 cm in men > 88 cm in women; MACE: major adverse cardiovascular events; CHD: coronary heart disease; PAD 3-4: peripheral arterial disease, stage 3 or 4 according to the classification of Leriche and Fontaine; CHF: congestive heart failure.
