# Supplementary material for: Metabolic syndrome in haemodialysis patients: prevalence, determinants and association to cardiovascular outcomes
Source: BMC Nephrol. 2020 Aug 13;21:343. doi: 10.1186/s12882-020-02004-3 (PMC7427285; doi:10.1186/s12882-020-02004-3)
Supplement: Supplementary file 4 — Additional file 4 Figure s1. Forest plots of MetS, MetS without BPH, and cumulative effect of components of the metabolic syndrome in predicting MACE in multivariate analysis. [file 12882_2020_2004_MOESM4_ESM.docx]

**Figure s1: Forest plots of MetS, MetS without BPH, and cumulative effect of components of the metabolic syndrome in predicting MACE in multivariate analysis.**

MetS: metabolic syndrome; MetS without HBP: MetS without considering high blood pressure; MsWC: waist circumference > 102 cm in men > 88 cm in women; MsHBP: history of hypertension or ongoing antihypertensive treatment; MsHDL: HDL cholesterol < 0.50 g/L in women, < 0.40 g/L in men; MsTG: TG ≥1.5 g/L; MsGlc: fasting glucose ≥1.00 g/L.
